# Development of a Triple Gene *Cry1Ac*-*Cry2Ab*-*EPSPS* Construct and Its Expression in *Nicotiana benthamiana* for Insect Resistance and Herbicide Tolerance in Plants

**DOI:** 10.3389/fpls.2017.00055

**Published:** 2017-01-24

**Authors:** Rubab Z. Naqvi, Muhammad Asif, Muhammad Saeed, Shaheen Asad, Asia Khatoon, Imran Amin, Zahid Mukhtar, Aftab Bashir, Shahid Mansoor

**Affiliations:** ^1^Agricultural Biotechnology Division, National Institute for Biotechnology and Genetic EngineeringFaisalabad, Pakistan; ^2^Pakistan Institute of Engineering and Applied SciencesNilore, Pakistan; ^3^Department of Biological Sciences, Forman Christian CollegeLahore, Pakistan

**Keywords:** Cry1Ac, Cry2Ab, EPSPS, *Nicotiana benthamiana*, plant transformation, armyworm, bioassay

## Abstract

Insect pest complex, cotton leaf curl disease and weeds pose major threat to crop production worldwide, including Pakistan. To address these problems, in the present study a triple gene construct harboring *Cry1Ac*, *Cry2Ab*, and *EPSPS* cassettes has been developed for plant specifically in cotton transformation against lepidopteron insect-pests and weeds. *Nicotiana benthamiana* (tobacco) was used as a model system for characterization of this triple gene construct. The construct has been assembled in plant expression vector and transformed in *N. benthamiana.* In six transgenic tobacco lines the integration of *Cry1Ac-Cry2Ab*-*EPSPS* in tobacco genome was checked by PCR, while successful protein expression of all the three genes was confirmed through immunostrip assay. Efficacy of *Cry1Ac* and *Cry2Ab* was evaluated through insect bioassay using armyworm (*Spodoptera littoralis*). These transgenic tobacco plants showed significant insect mortality as compared to control plants during insect bioassay. Three out of six tested transgenic lines L3, L5, and L9 exhibited 100% mortality of armyworm, while three other lines L1, L10, and L7 showed 86, 80, and 40% mortality, respectively. This construct can readily be used with confidence to transform cotton and other crops for the development of insect resistant and herbicide tolerant transgenic plants. The transgenic crop plants developed using this triple gene construct will provide an excellent germplasm resource for the breeders to improve their efficiency in developing stable homozygous lines as all the three genes being in a single T-DNA border will inherit together.

## Introduction

Cotton is a leading fiber crop in the world. It is a major cash crop in Pakistan that contributes about 60% in foreign exchange. Cotton provides raw material for textile industry and is considered as the backbone of economy. It makes up to 8.2% of the value added in agriculture and about 2% to GDP. In Pakistan, cotton crop provides source of income to millions of people, however, its production is progressively becoming expensive. Insect pest complex, cotton leaf curl disease (CLCuD) and weeds are mainly responsible for low productivity of cotton (Aslam et al., 2004).

Insects are a major threat to cotton production in Pakistan. The annual losses in cotton crop due to insect pests in Pakistan have been reported up to 2.5 million bales (Arshad et al., 2009). The insect pests of cotton are divided into chewing and sucking insects. Chewing insects mainly include cotton bollworm (*Helicoverpa armigera*), pink bollworm (*Pectinophora gossypiella*), spotted bollworm (*Earias insulana*), and armyworm (*Spodoptera littoralis*) (Babar et al., 2013). Sucking insects mainly include whitefly (*Bemisia tabaci*), thrips (*Thrips tabaci*), jassid (*Amrasca devastans*), and aphids (*Aphis gossypii*). Recently several other sucking pests such as mealybug (*Phenacoccus solenopsis*) and dusky bug (*Oxycarenus laetus*) have appeared on cotton crop. These destructive pests considerably reduce cotton yield by causing serious damage to this important crop. Sucking pests cause damage by sucking the sap from phloem tissues or by acting as vectors to transmit viral diseases (Ali and Aheer, 2007). Whitefly serves as a vector for CLCuD which is a major threat to cotton crop in Pakistan (Mansoor et al., 2006).

Insect pest management mainly relies on chemical pesticides to control these cotton pests. Almost 23% of all the insecticides used annually are for cotton on a global basis, however, most of these are found to be toxic and carcinogenic (James and Krattiger, 1997). Non-judicious and repeated use of these insecticides may result in development of resistance in target insect pests (Ishtiaq and Saleem, 2011). Such repeated use of pesticides has caused direct as well as indirect harmful effects on non-target insects, human health and overall environment which has led to the exploration of alternative options (Abdelgadirand and Adam, 2011).

Weeds are another major factor that affect cotton yield on an average by 37% (Ali et al., 2013). Weeds are very efficient users of resources and compete with the cultivated crop for water, sunlight, and other available nutrients and also provide shelter and food for insect pests and plant pathogens. Control of weeds is crucial to increase the cotton yield. Integrated weed management approaches including manual hoeing, mechanical methods and chemical or herbicide spray are emphasized for controlling weeds in cotton. Combination of mechanical weed control including manual weed hoeing is a time consuming and labor intensive way of removing weeds.

Up till now, many strategies have been devised for insect resistance development in high yielding cotton varieties against insect pests. Cotton improvement through conventional breeding for incorporation of insect resistance from existing germplasm also met with little success due to the non-availability of insect resistance genotypes. Problems associated with hazardous pesticides, herbicide and lack of desired traits in cotton germplasm can be addressed by application of biotechnology. Various insect resistance genes have been introduced into cotton using genetic engineering to provide better protection against insect pests (Vajhala et al., 2013). Different studies have shown the development of single gene or double gene constructs for *bt* genes developed under 35S promoter for cotton transformation. Single gene constructs for herbicide tolerance gene are also reported to be cloned for transformation purposes (Chen et al., 2006; Bakhsh et al., 2012; Muzaffar et al., 2015; Puspito et al., 2015).

Bt crops that harbor genes from *Bacillus thuringiensis* have demonstrated their effectiveness as alternatives to the synthetic insecticides (Raybould and Quemada, 2010). In a survey it was found that first generation Bt cotton’s adoption has raised farmer’s profit mostly by decreasing the pesticides expenditures and ultimate losses due to pest attack (Nazli et al., 2010). Although, Bt toxins are safe for non-target insects, their narrow spectrum of activity makes them less effective against a number of target insects.

Benefits of Bt crops expressing Cry proteins are enormous, like reduced use of synthetic insecticides, improved yield, higher income, lower production costs and compatibility with integrated pest management program. However, without adopting appropriate resistance management strategies, the effectiveness of Bt crops may be short. For better protection against target insect pests, the expression of a toxic protein should be sufficient enough in potentially vulnerable parts and at desired growth stage of the plant. High dose and refuge strategies for delaying insect resistance to Bt toxin have been effectively employed in the developed countries. But in Pakistan the situation is different because most of the Bt cotton varieties approved for general cultivation have comparatively low expression level of toxin (Ullah et al., 2014). Even most of farmers do not follow appropriate refugia plants and there is a growing concern that planting of Bt-cotton expressing low levels of toxin may limit its efficacy.

Resistance to the Bt toxin *Cry1Ac* in *H. armigera* is considered the major threat to the long-term effectiveness of Bt crops. Interaction of Bt Cry toxins with insect midgut epithelial receptors is a vital determinant of toxin specificity and insect resistance (Bravo et al., 2007). This issue of resistance development in target insects can be addressed by developing transgenic cotton expressing more than one toxin in plants like *Cry1Ac* and *Cry2Ab* for durable resistance against broad range of insect pests (Sohail et al., 2012).

As far as weed control is concerned, biotechnology also offers a better alternative to the conventional methods of weed control. Glyphosate (*N*-phosphonomethylglycine), commercially known as “Roundup” is the most widely used and relatively cheaper herbicide. It is a potent herbicide which is capable of stop ping the growth of a broad range of crops and weeds (Schmid and Amrhein, 1995). Glyphosate interferes in the shikimate metabolic pathway by inhibiting synthesis of 5-enolpyruvyl-3-phosphoshikimate (*EPSPS*). This prevents the succeeding synthesis of the three aromatic amino acids, phenylalanine, tryptophan, and tyrosine and is subsequently lethal to plants (Herrmann and Weaver, 1999).

The present study is aimed to assemble a triple gene *Cry1Ac*, *Cry2Ab*, and *EPSPS* cassette in a binary vector and its transformation in model plant (*Nicotiana benthamiana*). This construct has been designed for cotton transformation against lepidopteron insect-pests and glyphosate. Since cotton transformation is a long procedure, therefore, *N. benthamiana* (tobacco) was used as a model system for characterization of this triple gene construct. These three genes may result in the development of insect resistance as well as herbicide tolerance in *N. benthamiana*. This assembled triple gene construct gives an opportunity to speed up the development of cotton varieties conferring insect resistance and herbicide tolerance.

## Materials and Methods

### Plasmid Construction

Cry1Ac, Cry2Ab, and EPSPS gene sequences were retrieved from NCBI. The gene sequences were codon optimized according to upland cotton [*Gossypium hirsutum* [gbpln]: 557 CDS’s (190383 codons)] and were synthesized commercially. 2X 35S promoter (875 bp), Cry1Ac gene (1.876 Kb) and 35S terminator (773 bp) were amplified using specific primers (**Table 1**). All the PCR amplifications were carried out using *pfu* DNA polymerase. The 2X 35S promoter was independently cloned in T-Zero (yielding the vector T-2X35S), while a separate construct was made in T-Zero for Cry1Ac and 35S terminator (yielding the vectors T-Cry1Ac and T-35ST respectively). The 2X 35S promoter was cloned first in pBlue ScriptSK-Zero (from the clone in T-2X35S) using the *Swa*I and *Bam*HI restriction sites (yielding the vector pBlueScriptSK-2X35S). The Cry1Ac gene was cloned from T-Cry1Ac in the pBlueScriptSK-2X35S using *Bam*HI and *Hind*III restriction sites. The terminator was cloned using the *Hind*III and *Sal*1 restriction sites.

**Table 1:** Primer Sequences used in triple gene plasmid construction.

Primer name	Primer sequences	Product
35SF	5′ CGCATTTAAATCTTAATTAATCCCCAGATTAGCCTTTTCAATTTCAG 3′	2X 35S promoter
35SR	5′ ATAGGATCCAGCTTGTCAGCGTGTCTCTCCAAATGAAATGAAC 3′	
Cr1AcF	5′ CTAGGATCCAGCAGATCGAAATGGACAATAATCCGAATA 3′	*Cry1Ac* gene
Cr1AcR	5′ GCTAAGCTTCTAGATCATTCTAAAGTTGCAGTAACTG 3′	
35STF	5′ GTTAAGCTTACCGTCACCGGTGTGAGGGAACTAG 3′	35S terminator
35STR	5′ TATGTCGACACCTAAGGTTCGGACGGTACGCTGAA 3′	
2AbF	5′ TCTGGATCCACAAATCTCATGCAAGATATCC 3′	*Cry2Ab* gene and E9 terminator
G7R	5′ CAGAAGCTTCGCGTCGACGATCTTGAAAGAAATATAG 3′	
CVMF	5′ CTCATTTAAATGAAGGTAATTATCCAAGATGTAGCATCAAG 3′	CVM promoter
CVMR	5′ TTCGGATCCTTCACCACAAACTTACAAATTTCTC 3′	
*EPSPS*F	5′ GAAGGATCCGAAATGGCTCAAATAAACAACATGGCT 3′	*EPSPS* gene and E9 terminator
E9R	5′ ATTAAGCTTGGCGCGCCGATGTTTTACTCCTCATAT 3′	

The partial Cry2Ab and the G7 terminator (1.88 kb) was amplified using specific primer pair (**Table 1**). This fragment was cloned first in T-Zero using the *Bam*HI and *Hind*III restriction sites (yielding the vector T-Cry2Ab-G7). The Cry2Ab-G7 cassette was cloned from T- Cry2Ab-G7 in the pBlue Script SK-Zero using *Bam*HI and *Hind*III restriction sites to obtain the vector pBlueScriptSK-Cry2Ab-G7. The figwort mosaic virus (FMV) promoter, chloroplast signal peptide and partial Cry2Ab (270 bp) was commercially synthesized and cloned independently in pre-developed pBlueScriptSK-Cry2Ab-G7 using restriction enzymes *Bam*HI and *Swa*I. This yielded the vector pBlueScriptSK-FMV-Signal EPSPS-Cry2Ab-G7. Finally, verification of the complete cassette in pBlueScriptSK was done by restriction analysis.

The cassava vein mosaic virus (CVM) promoter (700 bp), EPSPS-E9 terminator (1.9 Kb) were PCR amplified with pfu DNA polymerase using specific primers (**Table 1**). The CVM promoter was independently cloned in T-Zero (yielding the vector T-CVM), while a separate construct was made in T-Zero for EPSPS-E9 terminator (yielding the vector T-EPSPS-E9). The CVM promoter was cloned first in pBlueScriptSK-Zero (from the clone in T-CVM) using the *Swa*I and *Bam*HI restriction sites (yielding the vector pBlueScriptSK-CVM). The EPSPS-E9 cassette was then cloned from T-EPSPS-E9 in the pBlueScriptSK-CVM using *Bam*HI and *Asc*I restriction sites.

**FIGURE 1:**
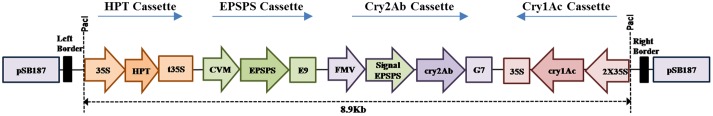
**Construct map of pBT-*Cry1Ac*-*Cry2Ab*-*EPSPS***.

As the three cassettes were ready in pBlueScriptSK, following strategy was used to assemble the gene construct in binary vector pSB187 (having rare cutter sides added) stepwise. FMV-Signal EPSPS-Cry2Ab-G7 cassette was cut from pBlueScriptSK-FMV-Signal EPSPS-Cry2Ab-G7 using *Sgr*DI and cloned in the *Sgr*DI site of pSB187. Afterward, cutting of the 2X35S-Cry1Ac-35S from plasmid pBlueScriptSK-2X35S-Cry1Ac-35S was done using Eco811 and Pac1, and cloned in the respective sites of pSB187 having the FMV-Signal EPSPS-Cry2Ab-G7 cassette. Finally, the CVM-EPSPS-E9 cassette was cut from plasmid pBlueScriptSK-CVM-EPSPS-E9, using Swa1 and Asc1, and cloned in the respective sites of pSB187 having the FMV-Signal EPSPS-Cry2Ab-G7 and 2X35S-Cry1Ac-35S cassettes (**Figure 1**). The final triple gene construct Cry1Ac-Cry2Ab-EPSPS in pSB187 (**Figure 1**) was ultimately transformed to *Agrobacterium tumefaciens* strain AGL1 competent cells for stable integration into the plant genome through tissue culture techniques.

### Transient Expression of Triple Gene Construct

Plasmid pBT-*Cry1Ac*-*Cry2Ab*-*EPSPS* was transformed into *A. tumefaciens* strain AGL1 through electroporation. Afterward, *Agrobacterium* culture was prepared and agroinfiltrated into *N. tabacum* cv. Samson and *N. benthamiana* leaves as described earlier (Leckie and Stewart, 2011). Three leaves each of the five plants were infiltrated with *Agrobacterium* suspension. Samples were collected after 72 h, then expression of *Cry1Ac* and *Cry2Ab* genes was confirmed by using immunostrip test (Cat No. AS012LS Envirologix, USA).

### Stable Transformation of Tobacco for Characterization of Triple Gene Construct

*Agrobacterium tumefaciens* strain AGL1 having pBT-*Cry1Ac*-*Cry2Ab*-*EPSPS* plasmid was used for transformation of *N. benthamiana*. Leaf disc (10 mm) were cut from aseptically grown *N. benthamiana* plants and was placed on MS0 medium (Murashige and Skoog, 1962). Plates were wrapped and incubated at 16/8 light and dark cycle at 25 ± 1°C temperature. Bacterial culture was grown from a single colony on a freshly streaked plate. After 48 h, *Agrobacterium* suspension was poured into a sterile Petri plate. All leaf discs were placed in bacterial suspension for 15–20 min, with gentle shaking at regular intervals. Leaf discs were blot dried on sterile filter paper and were transferred to co-cultivation medium that is MS0 medium having 1-naphthaleneacetic acid 0.1 mg/l and 6-benzylaminopurine 1 mg/l. Plates were sealed with Parafilm and kept at 25 ± 1°C for 48 h under light conditions. After 2–3 days on co-culture medium, the leaf discs were removed and washed with liquid MS0 medium having cefotaxime (250 mg/l). After washing, the leaf discs were blot dried and placed on selection medium (MS0 medium having cefotaxime 100 mg/l and hygromycin 30 mg/l) at 25 ± 1°C for 2 weeks. Leaf discs were transferred to fresh selection medium every 15 days. The regenerated plants having shoots with approximately length of 1 cm, were transferred to fresh medium in magenta containers. Plants with well-established shoots were separated and transferred to MS0 medium without growth regulators for rooting.

### Molecular Analysis of Putative Transgenic Plants

Genomic DNA of putative transgenic plants was extracted and then subjected to PCR analysis for confirmation of Cry1Ac, Cry2Ab, and EPSPS triple gene construct in plant genome. A reaction mixture of 25 μl was prepared by adding 5 μl of 50 ng plant DNA, 2.5 μl of 10X PCR buffer, 2.5 μl dNTPs (2 mM), 1 μl MgCl_2_ (25 mM), 0.5 μl of each gene specific primer (**Table 2**), 0.25 μl Taq DNA polymerase (Thermo Fisher Scientific) and deionized water to make up the remaining volume. Reaction was held in a PCR tube and incubated in a thermocycler, programmed as for 5 min preheat at 95°C and then 35 cycles of having denaturation at 94°C for 1 min, annealing temperature of 52°C for 1 min and extension of 1 min at 72°C, with a final extension temperature 72°C for 10 min. Expression of transgenes at protein level was confirmed by Immunostrip test (Cat No. AS064 LS, Envirologix, USA). Two leaf punches from all the putative transgenic tobacco lines were collected independently. Each leaf sample was separately ground for 20–30 s in the pestle mortar. Grinding step was repeated after adding 250 ul of 1X Extraction buffer (provided with Cat No. AS064 LS, Envirologix, USA). This mixture was transferred to a sterile eppendorf tube and centrifuged for 2 min. The supernatant was transferred to a new sterile eppendorf tube and the immunostrip strip was placed into it. The sample traveled up the strip. The strips were allowed to develop bands for 5 min before making final assay interpretations.

**Table 2:** Primer sequences for triple gene construct transgene analysis.

Primer name	Primer sequence 5′–3′
Hyg-F	AGAATCTCGTGCTTTCAGCT
Hyg-R	ACATTGTTGGAGCCGAAAT
CR1BDR5	ATGTCCATAAGGTGAGGTG
CR1BDF5	TTGCGTGAAGAGATGAGG
CR2BDR4	ACTTGAGTGGCGTGTATG
CR2BDF4	CGGTGCTAACTTGTATGC
EPSR3	GCGAGACGGAGATTTATT
EPSF3	TGGGTTTGGTTGGTGTTT

### Insect Bioassays

Efficacy of Cry1Ac and Cry2Ab was checked through bioassays of detached leaf with first instar larvae of armyworm (*S. littolaris*). Leaves from putative transgenic plants were removed and placed on a moist filter paper in a Petri plate. Five first instar larvae were placed in on a leaf each plastic Petri plate. These plates were kept at 25 ± 1°C and 50–70% relative humidity. The insect bioassay was repeated twice along with controls, which consisted of leaves from non-transgenic tobacco plants. Insect mortality data was recorded using stereo-microscope after every 24 h till 5 days of the experiment. The data of insect bioassay was statistically analyzed using analysis of variance (ANOVA) and least significant difference test (LSD) to calculate the difference in insect mortality between transgenic and control plants.

## Results

### Construction of Plasmid Containing Triple Gene Construct

All the confirmed clones in pBluescript SK (+) were verified by PCR with respective primers and restriction analysis. Ultimately, a single plasmid was obtained successfully by cloning all the three gene cassettes in binary vector pSB187. A chloroplast transit peptide of *Arabidopsis thaliana* 5-enolpyruvyl shikimate-3-phosphate synthase gene was used with *Cry2Ab* gene cassette to direct the protein to the chloroplasts. The T-DNA border of the binary vector was having 35S: *hpt:*t35S for hygromycin resistance. The final triple gene construct was named as pBT-*Cry1Ac*-*Cry2Ab*-*EPSPS*, its structure is shown in **Figure 1**. The final triple gene construct (**Figure 1**) was ultimately transferred to *A. tumefaciens* strain AGL1 competent cells for tobacco transformation. The final triple gene cassette sequence is submitted to GenBank under accession no. KX880509.

### Transient Expression of Cry1Ac, Cry2Ab, and EPSPS

**FIGURE 2:**
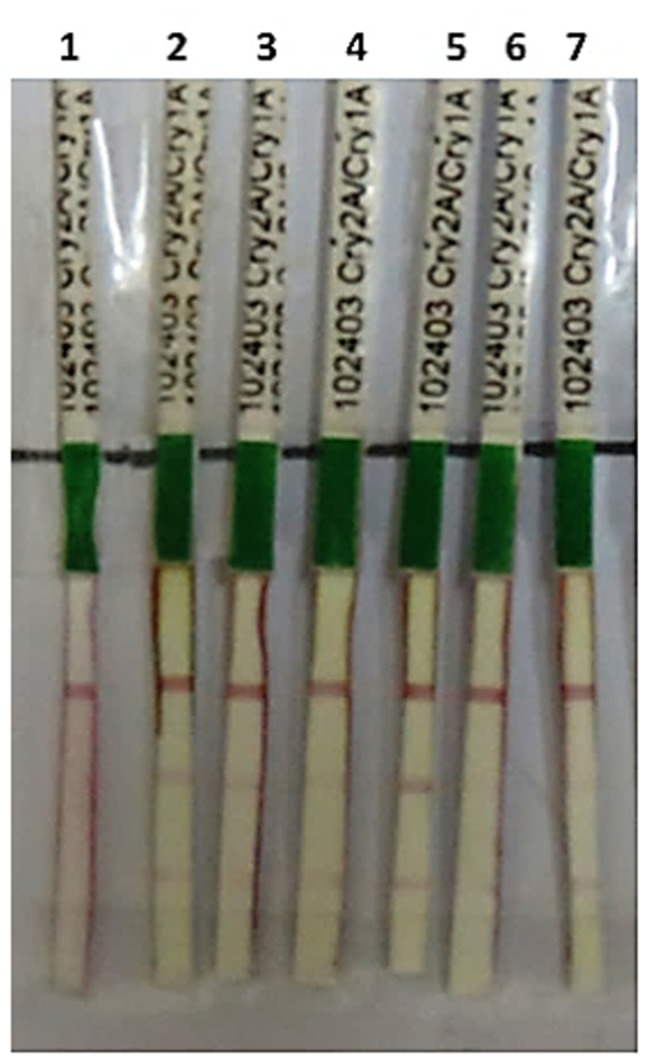
**Immunostrip test of *N. tabacum* and *Nicotiana benthamiana* plants transiently transformed with pBT-*Cry1Ac*-*Cry2Ab*-*ESPSP* construct: 1 = negative control, 2–4 = transiently transformed leaves of *N. benthamiana*, 5–7 = transiently transformed leaves of *N. tabacum***.

Transient expression analysis of a transgene is a good and early verification of gene expression before its stable transformation into a desired plant. Thus in the current study, before stable transformation of triple gene construct, it was transiently expressed in tobacco plant. Triple gene construct pBT-Cry1Ac-Cry2Ab-EPSPS was successfully expressed transiently in tobacco through agroinfiltration. During the transient assay, Cry1Ac and Cry2Ab proteins were detected after 72 h through immunostrip assay which indicated the successful expression of these genes in all the agroinfilterated tobacco leaves (**Figure 2**).

### Transformation of Triple Gene Construct in *N. benthamiana*

*Nicotiana benthamiana* was transformed with *A. tumefaciens* carrying pBT-*Cry1Ac*-*Cry2Ab*-*EPSPS* and independent lines were maintained. All putative lines showed normal plant growth and set viable seeds. About 50 leaf discs of *N. benthamiana* were transformed with *A. tumefaciens* having pBT-*Cry1Ac*-*Cry2Ab*-*EPSPS*, in two different experiments with overall transformation efficiency of 62%. The remaining explants bleached out with time on hygromycin selection medium. The regeneration of plantlets was observed after 35 days from the date of transformation. After complete shooting and rooting of plants, six transgenic lines were selected and maintained for further molecular analysis.

### Molecular Analysis of Putative Transgenic Plants

**FIGURE 3:**
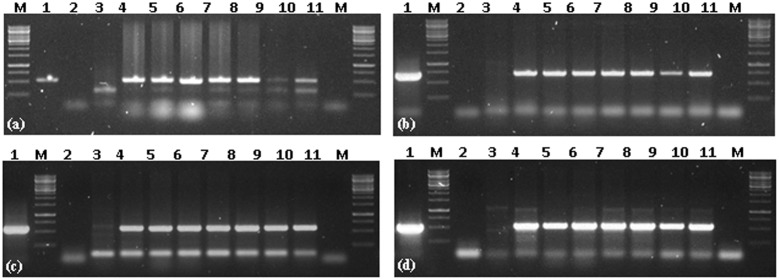
**Transgene PCR confirmation of *Cry1Ac-Cry2Ab-EPSPS* transgenic *N. benthamiana* plants. (a)** PCR amplification of *Cry1Ac*, **(b)** PCR amplification of *Cry2Ab*, **(c)** PCR amplification of *EPSPS*, **(d)** PCR amplification of *hpt*; *M* = 1 Kb Marker; 1 = positive control (Plasmid); 2 = negative PCR control; 3 = *N. benthamiana* control plant; 4–10 = *Cry1Ac-Cry2Ab-EPSPS* transgenic *N. benthamiana* plants; 11 = negative PCR control.

**FIGURE 4:**
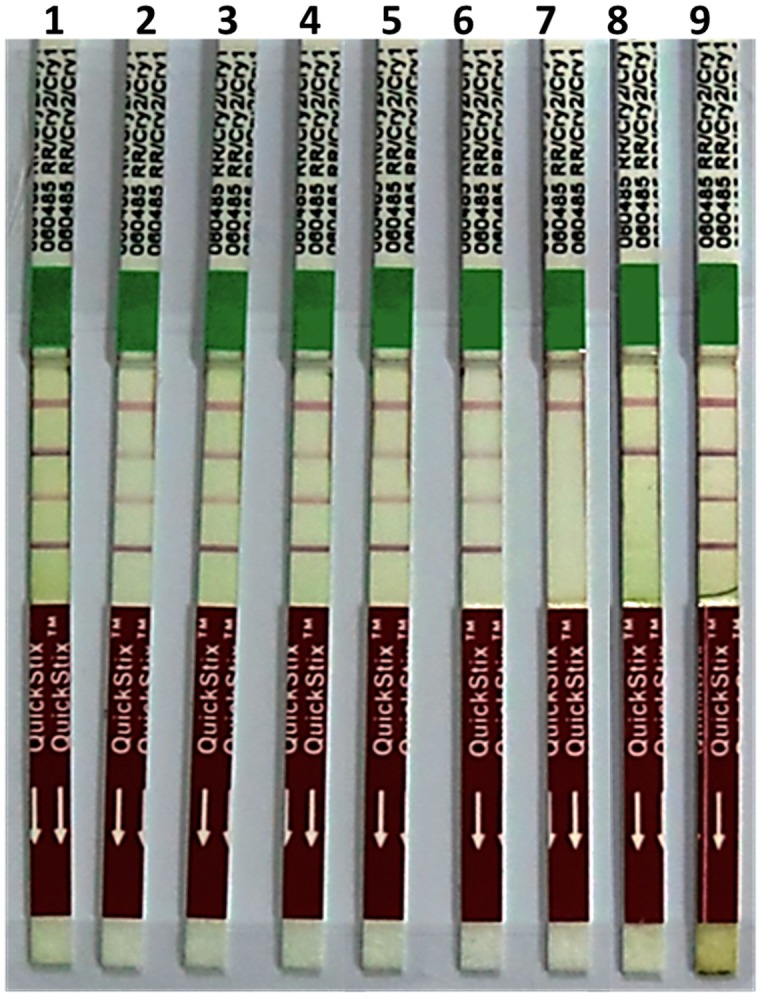
**Immunostrip test of *N. benthamiana* plants transformed with pBT-*Cry1Ac*-*Cry2Ab*-*EPSPS* construct (Immunostrip kit Cat No.** AS064 LS, Envirologix, USA): 1–7 = transgenic plants of *N. benthamiana;* 8–9 = negative controls of *N. benthamiana;* 10 = non-transgenic plant of *N. benthamiana* as a negative control, 11 = extraction buffer as a negative control; 12 = Mon531 IR cotton as a positive control for Cry1Ac; 13 = Monsanto Bollgard II plus Roundup Ready cotton as a positive control for Cry1Ac, Cry2Ab, and *EPSPS*.

All six putative transgenic plants selected on hygromycin showed amplification of 521, 614, 568, 510 bp fragments representing internal sequence of *Cry1Ac*, *Cry2Ab*, *EPSPS*, and *hpt* cassettes, respectively (**Figure 3**). Successful expression of three *Cry1Ac*, *Cry2Ab*, *EPSPS* genes at protein level was ensured through immunostrip assay in all six selected transgenic lines (**Figure 4**).

### Insect Bioassays

**FIGURE 5:**
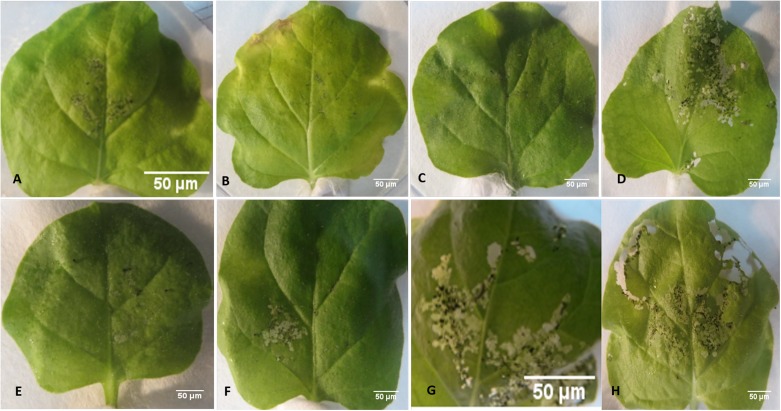
**Insect bioassay of pBT-*Cry1Ac*-*Cry2Ab*-*EPSPS N. benthamiana* transgenic plants with *Spodoptera littoralis*. (A)** Transgenic line 1, **(B)** transgenic line 3, **(C)** transgenic line 5, **(D)** transgenic line 7, **(E)** transgenic line 9, **(F)** transgenic line 10, **(G,H)** *N. benthamiana* control lines. Scale bars **(A–H)**: 50 μm.

**FIGURE 6:**
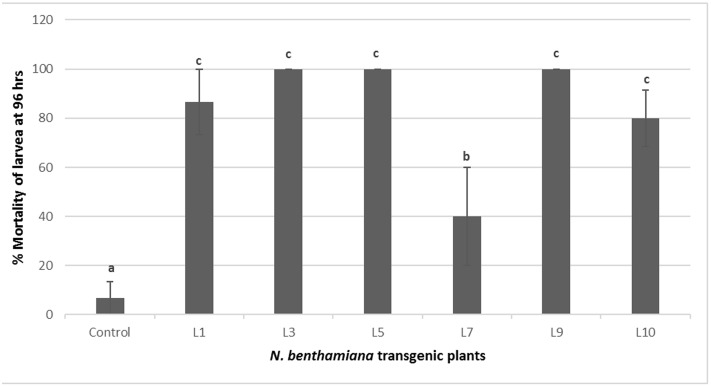
**Percent mortality of *S. littoralis* in insect bioassay of *N. benthamiana* transgenic plants harboring *Cry1Ac*-*Cry2Ab*-*EPSPS*.** Control: *N. benthamiana* non-transgenic control plant, L1-L9: *N. benthamiana* transgenic plants. Different letters on the top of bars indicate significant differences between transgenic lines and control at *P* ≤ 0.05.

The transgenic tobacco lines positive for PCR and showing *Cry1Ac, Cry2Ab* expression were selected for insect bioassays. These transgenic tobacco plants showed significant insect mortality as compared to control plants during insect bioassay. Three out of six tested transgenic lines L3, L5, and L9 exhibited significant armyworm mortality up to 100%, while three other lines L1, L10, and L7 showed 86, 80, and 40% mortality, respectively. Only one PCR positive line L7 showed less insect mortality, i.e., 40% during the bioassay. Various transgenic events tested for the insect bioassays and percentage insect mortality is shown in **Figures 5** and **6**, respectively.

## Discussion

For the sake of sustainable agriculture, biotechnology offers environment friendly approaches to manage insect pests and weeds. The most effective method is to isolate and develop gene constructs for insect resistance and herbicide tolerance to produce transgenic crops. In the present study the triple gene *Cry1Ac*-*Cry2Ab*-*EPSPS* construct has been designed and assembled for the transformation in cotton to develop transgenic cotton with insect resistance and herbicide tolerance. Before cotton transformation, this assembled *Cry1Ac*-*Cry2Ab*-*EPSPS* construct is evaluated in tobacco model system.

The coordinated delivery and manipulation of multiple desired traits into plants presents a unique challenging task for molecular biologists. One of the best approaches is the transformation of plants by delivering multiple gene cassettes cloned in a single vector. Delivering multiple gene cassettes by a single vector is advantageous over the use of multiple vectors as only a single DNA molecule needs to be transferred into the cells for better transgene expression. Thus, a smaller number of plants typically need to be generated, as compared to other methods like retransformation by using multiple vectors. Furthermore, as a single DNA molecule will integrate into the plant genome, therefore, all the genes cloned in a single T-DNA will most likely be inherited together (Halpin and Boerjan, 2003; Dafny-Yelin and Tzfira, 2007).

Previously Monsanto has developed insect resistant and herbicide tolerant cotton that is a product of traditional plant breeding of insect-resistant cotton with the herbicide-tolerant cotton. To our knowledge the present study is the first effort to develop a vector having triple gene construct for insect resistance and herbicide tolerance. This triple gene construct will provide an opportunity to the breeders for crop improvement by developing homozygous lines, as triple genes will co-inherit in the next generations.

The *hpt* gene codes for hygromycin phosphotransferase (HPT), which detoxifies the antibiotic hygromycin B (Wang and Waterhouse, 1997). Many plants have been transformed with the *hpt* gene and hygromycin B has proved to be very effective and efficient in the selection of a wide range of transgenic plants including tobacco and cotton (Harrison et al., 2006; Bibi et al., 2013). In the current study hygromycin is used as a plant selection marker for two main reasons. One is to have an efficient selection of transgenic plants and second one is the replacement of kanamycin that is most widely used antibiotic for plant selection in tissue culture. Thus, in the long run to use this construct in the cotton transformation, hygromycin selection will help in re-transformation and to avoid false positives by the selection based on kanamycin resistance.

Insects are more prone to toxins at early stage of their life and high level of toxin expression in plants contribute toward their efficient control. So to uphold a certain level of toxin in plants, a toxin encoding gene must be expressing continuously. By optimization of some important factors such as codon usage bias, GC content and mRNA secondary structure, higher expression levels of exogenous or transgenes can be achieved (Liu et al., 2004). For the development of multiple gene cassettes in a single vector, the higher mRNA transcripts of each target gene can be generated independently by using separate promoters in front of every gene. Therefore, the resulting vector leads to an increased expression of each of the protein encoded by its own promoter as compared to clone all the genes under one promoter (Mitsuhara et al., 1996; Kim et al., 2004). In this study, the triple genes were codon optimized and cloned under three different constitutive promoters and terminators. CVM promoter and FMV promoter are also known to have strong expression levels of transgenes analogous to 35S promoter (Verdaguer et al., 1996, 1998).

In the present study, the toxins Cry1Ac and Cry2Ab expressed under 2X35S and FMV promoters respectively have considerably high expression levels that was enough for insect mortality. This was confirmed by conducting insect bioassays using *S. littoralis* on the putative transgenic plants. Most of the selected lines showed considerably high mortality up to 100% of *S. littoralis* larvae after 5 days of the insect bioassay. One of the transgenic lines showed little (40%) resistance to insect during bioassays. However, it was observed that insect size was not increased while feeding on the leaves of this particular transgenic line comparable to control lines where insects become healthy after feeding on leaves. This variation in expression levels could be due to many factors including the positional effect of the integrated gene, recombination or rearrangement before transgene integration, transgene copy number and DNA methylation (Meyer, 1998). Hence integration site of transgene in the plant genome may lead to a detrimental or negative effect on its expression. Methylation of gene in plant genome can result in partial or complete silencing of the foreign gene. Plant with low level of resistance may have suffered by methylation of transgenes by which expression of these genes was poor (Matzke and Matzke, 1991).

In previous studies, synergistic effects of *Cry1Ac* and *Cry*2Ab toxins expressing in tobacco have shown high mortality rates in both *H. armigera and Spodoptera exigua* larvae. It shows that these both toxins have different binding sites, indicating their potential usefulness for insect control in transgenic cotton (Ibargutxi et al., 2008; Sohail et al., 2012). *Cry1Ac* and *Cry*2Ab toxins used in current study will provide a more effective insect resistance management program compared to single gene products. In addition to effective insect control, this combination of toxins may help to delay the development of resistance to Bt toxins among insect population.

Weeds are another major factor that affect cotton yield. Weeds very efficiently compete with the cultivated crop for water, sunlight, and other available nutrients and also provide shelter and food for insect pests and pathogens. Weed control in cotton by integrated weed management approaches including manual hoeing, chemical, or herbicide spray is time consuming and labor intensive (Awan et al., 2015). Here, in the present study we have introduced *EPSPS* successfully in the triple gene vector for herbicide tolerance, which will help the farmers in future for the control of weeds in cotton. A previous study (Puspito et al., 2015) has shown the efficacy of similar genes for insect and herbicide tolerance using co transformation of different vectors in cotton. Our results support the conclusion of previous studies (Awan et al., 2015; Puspito et al., 2015) and additionally provide novel insights by introducing triple gene construct in a single T-DNA. This triple gene construct will allow the breeders to improve their efficiency for developing homozygous lines as all the three genes being in a single T-DNA border will integrate in genome and inherit together.

In nutshell, newly developed triple gene *Cry1Ac-Cry2Ab*-*EPSPS* construct showed significant insect mortality and protein expression of all the three genes in transgenic plants. Hence, it could be transformed further in cotton or other crop plants to obtain cotton or other crop varieties with improved insect control. Furthermore, the construct provides plants with herbicide tolerance up to certain limits of glyphosate, that may be elevated in future by opting Roundup Ready Flex technology for superior and effective broad spectrum weed control.

## Author Contributions

SM, AB conceived and designed the current project. AB, ZM, MS, IA performed vector designing of single gene constructs. AB designed the triple gene construct and its cloning strategy. AK, RN performed cloning experiments. MA contributed critically in expression analysis studies, statistical data analysis and interpretation of the results. MS, SA were involved in transformation experiments. RN performed transient expression analysis, transgene PCR analysis, expression analysis experiments with immunostrip, insect bioassay, interpretation of results and wrote this manuscript. MA, SM revised the manuscript critically.

## Conflict of Interest Statement

The authors declare that the research was conducted in the absence of any commercial or financial relationships that could be construed as a potential conflict of interest.
